# Forest tree diversity is dependent on both mycorrhizal type and scale

**DOI:** 10.1371/journal.ppat.1014112

**Published:** 2026-04-20

**Authors:** Kathryn N. Maley, Mary Catherine Aime

**Affiliations:** Department of Botany and Plant Pathology, Purdue University, West Lafayette, Indiana, United States of America; University of Tübingen: Eberhard Karls Universitat Tubingen, GERMANY

## Abstract

The diversity of forest communities is intricately shaped by the mycorrhizal associations that trees form with soil fungi. Mycorrhizae, which are mutualistic relationships between fungi and plants, play pivotal roles in nutrient exchange and tree survival. This review explores how the type of mycorrhizal association—arbuscular mycorrhizal (AM) or ectomycorrhizal (ECM)—impacts forest tree diversity across various scales. AM fungi dominate tropical forests, potentially contributing to high species richness, while ECM fungi are more prevalent in temperate regions, where they often correlate with lower diversity. We discuss how these associations influence soil properties, plant-soil feedback, and seedling establishment, ultimately shaping forest structure. We also examine the complexity of the relationship between mycorrhizal type and tree species richness, noting the differences in patterns observed across different spatial scales. As research continues, it will be crucial to examine local dynamics in underrepresented regions and explore how mycorrhizal interactions change over time to understand their role in maintaining forest biodiversity. By considering these dynamics, we can better predict how forests will respond to environmental changes and develop strategies for preserving and restoring forest ecosystems.

## “Mycorrhiza” = “fungus-root”

Mycorrhizae are widespread symbiotic relationships between fungi and plants. These associations are present in 80% of all terrestrial plant species and 92% of plant families [1]. Specialized soil fungi inhabit the roots of compatible host plants, forming structures for nutrient transfer through synchronized development [2]. Most mycorrhizal associations are balanced, with reciprocal exchanges between the plant and fungal partners [2]. Mycorrhizal fungi aid in water and nutrient uptake, while plants provide carbon-rich photosynthates [3,4]. Arbuscular mycorrhizal (AM) and ectomycorrhizal (ECM) symbioses are the two main types associated with trees [1].

**Fig 1:**
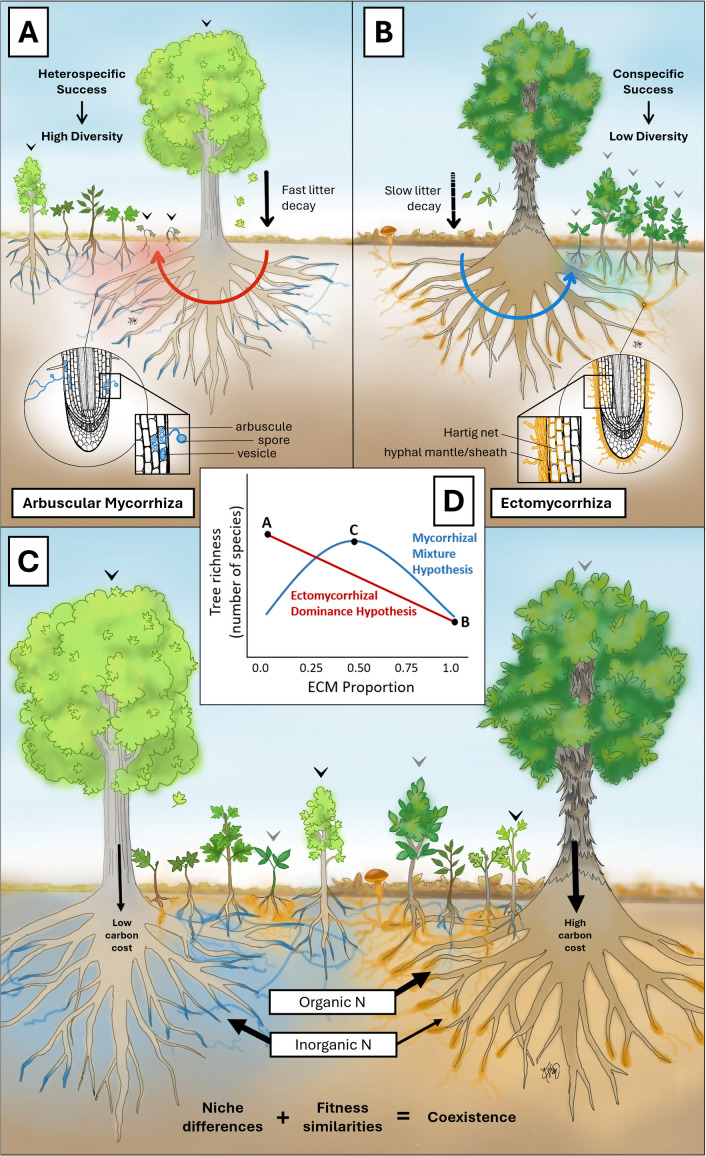
Hypothetical relationships between mycorrhizal types and tree species richness. Panels **A**, **B**, and **C** correspond to points A, B, and C on the graph in Panel **D**, which shows relationships between ECM tree proportion and tree species richness. Red arrows indicate negative feedback, and blue arrows show positive feedback. Trees colonized by AM fungi have blue root tips, while those colonized by ECM fungi have orange root tips.

AM fungi penetrate root cells and form nutrient transfer structures called arbuscules [2], while ECM fungi grow between and around root cells, forming a Hartig net and hyphal sheath or mantle [5] (Fig 1A versus 1B). Although these structures define a fungus as AM or ECM, mycorrhizal types also align with fungal phylogeny. AM fungi belong to the phylum Glomeromycota [6], while most ECM fungi belong to Basidiomycota.

## Evolutionary histories of AM and ECM fungi

Phylogenetic and palaeobotanical evidence suggests AM fungi evolved at least 255 million years before ECM fungi [7]. AM fungi evolved alongside land plants approximately 460 million years ago [8], playing a key role in the transition of plants from water to land by providing access to soil nutrients, enabling them to compete with soil microbes [7]. Today, AM fungi have a wide host range of over 200,000 plant species [9] despite being relatively species-poor with estimates ranging from 200 to 1,200 species in the Glomeromycota [10,11].

ECM fungi evolved independently from saprotrophic ancestors in several Basidiomycota and some Ascomycota and Mucoromycota taxa [12,13]. Some ECM fungi retain saprotrophic abilities, mining soil organic material for nutrients [14]. Many ECM fungi have reversed to saprotrophy after evolving ECM traits [13]. ECM fungi are more diverse than AM fungi, with some estimates as high as 25,000 species [15], but are host-specific to about 6,000 plant species from 30 lineages of angiosperms and gymnosperms, mostly woody perennials such as trees and shrubs [16].

## Global distributions of AM and ECM trees and fungi

Tree species typically follow a latitudinal diversity gradient [17], with the highest diversity in tropical regions and lower diversity toward the poles [18]. A forest is said to be AM- or ECM-dominated when a single mycorrhizal type occupies over 75% of tree basal area. Tropical forests are predominantly comprised of AM trees, while boreal forests are ECM-dominated [19]. Additionally, ECM fungi are most diverse in temperate regions [20], while AM fungi show consistent diversity across latitudes [10]. This trend is partially due to differences in nutrient accessibility: AM fungi are most efficient at inorganic nutrient uptake, while ECM fungi prefer organic nutrient forms [21]. Local factors such as host availability, dispersal limitation, and environmental conditions also affect AM fungi dynamics [22].

## AM and ECM trees’ impact on soil and seedling establishment

AM trees’ dominance in diverse forests may reflect a causal relationship in which they foster higher diversity (Fig 1A). AM trees often experience negative plant-soil feedback, meaning they alter their environment in ways that inhibit their own species’ growth [23]. This may occur through the attraction of species-specific herbivores and pathogens, forcing offspring to disperse farther, thus opening space for other species to establish [24]. This is consistent with the Janzen-Connell hypothesis, which was proposed in 1970 to explain high diversity in tropical forests [24,25].

In contrast, ECM trees generally experience positive plant-soil feedback, promoting their own species’ success and suppressing others [26]. This leads to low-diversity stands (Fig 1B), such as those common in boreal forests, although ECM-dominated forests can also occur in tropical regions [27]. Saplings of both AM and ECM trees grow faster and more abundantly near trees of the same mycorrhizal type, likely due to greater fungal partner availability and more accessible nutrients [28].

Mycorrhizal fungi can connect multiple trees through their mycelium, facilitating nutrient and chemical signal transfer. Saplings benefit from being linked to an existing mycorrhizal network, as nutrient flow follows source-sink dynamics [29]. However, the direction and strength of nutrient transfer depend on the plant and fungal partners involved [30].

## Mycorrhizal type and tree species richness at different scales

Research on the link between mycorrhizal type and tree species richness shows that AM and ECM trees influence the soil nutrient economy and forest structure differently [31]. The ECM dominance hypothesis suggests that species richness decreases as ECM trees increase (Fig 1D). This is supported by studies in global [32] and temperate regions [33], although studies in United States (U.S.) forests show conflicting results. A reciprocal transplant study in the U.S. indicated that AM seedling survival improved with heterospecific pairings, while ECM seedling survival was enhanced by conspecific pairings [34]. However, an observational study found a hump-shaped relationship between ECM proportion and species richness across U.S. forests, with peak richness in forests with equal AM and ECM proportions [35]. These results led to the mycorrhizal mixture hypothesis, which posits that approximately equal mixtures of AM and ECM trees promote species richness, while dominance by either type decreases richness (Fig 1D).

Niche partitioning of mineral nutrients could explain this pattern [36]. AM fungi primarily use inorganic nitrogen, while ECM fungi use organic nitrogen [31,37]. In environments with both forms of nitrogen limited, mixed AM and ECM forests could reduce competition for nutrients and support greater species diversity [37] (Fig 1C).

The ECM dominance hypothesis and mycorrhizal mixture hypothesis are not mutually exclusive. Both suggest decreased species richness with increasing ECM tree proportion (50%–100%), but they differ in the expected relationship at low and intermediate ECM proportions. Some regions, such as the U.S., may support the highest tree diversity with a mixture of both mycorrhizal types, while globally, AM-dominated forests tend to be more diverse. Local conditions may influence whether mixed mycorrhizal forests or AM forests are more diverse.

Different ecological mechanisms may shape diversity at various scales. At fine scales, niche complementarity between AM and ECM trees may promote coexistence by enabling efficient nitrogen usage. However, larger spatial scales may overlook these fine-scale differences and lead to classifications that group forests as either AM- or ECM-dominated.

## Conclusions and future studies

Future studies should focus on local-scale forest dynamics in regions underrepresented in current mycorrhizal research, such as Russia, Africa, and East Asia [32]. These studies could clarify whether the hump-shaped relationship between mycorrhizal types and species richness is universal or unique to the U.S. Including regions with AM-dominated and mixed mycorrhizal forests is essential to elucidating this relationship since these mixtures have been prone to discrepancies across scales. Though relatively rare worldwide, mixed mycorrhizal forests occur in the northeastern U.S., parts of southeastern Africa, and eastern Asia [19]. The global lack of mycorrhizal complementarity could be due to low sample sizes of mixed forests or the use of categorical data instead of continuous data that accounts for varying proportions of AM and ECM trees.

It is also crucial to account for temporal dynamics, as mycorrhizal dominance shifts throughout forest succession [28]. Global studies often combine data collected over long periods, from different researchers using varying methods, and do not always consider forest successional stages or forest age. It is vital that we consider these differences when making comparisons across different sites to elucidate the effects of mycorrhizal type on tree composition.

Because mycorrhizal type is closely correlated with climate [19], it is important to consider how climate change may affect this relationship. As the climate warms, conditions become more favorable for AM trees than ECM trees, a pattern that is evident in the increased AM-dominance in U.S. forests over the past three decades [38]. This is concerning because ECM biomass is linked to greater terrestrial carbon stocks than AM [39], suggesting that ECM trees mitigate climate change by reducing atmospheric carbon dioxide through efficient carbon sequestration. Of course, AM-dominance is linked to greater global tree diversity [21], which is important for maintaining forest resilience. It is imperative that we consider and balance these effects when selecting tree species for reforestation efforts or designating protected land in the future. Likely, the best combination of benefits would result from planting strategic mixtures of AM and ECM trees and protecting naturally occurring mixed mycorrhizal forests.

Mycorrhizal fungi are central to tree establishment and survival, and their influence on forest diversity is key to understanding forest dynamics. While the relationship between tree species richness and mycorrhizal type is complex and dynamic, it provides valuable insights into the mechanisms driving forest structure. Recognizing deviations from expected patterns can help identify shifts in forest structure, possibly due to anthropogenic factors or other changes. Understanding these processes is crucial for the development of effective conservation and restoration efforts in forests.
